# Supplementary material for: A microparticulate based formulation to protect therapeutic enzymes from proteolytic digestion: phenylalanine ammonia lyase as case study
Source: Sci Rep. 2020 Feb 27;10:3651. doi: 10.1038/s41598-020-60463-y (PMC7046617; doi:10.1038/s41598-020-60463-y)
Supplement: Supplementary file 1 — Supplementary information. [file 41598_2020_60463_MOESM1_ESM.pdf]

Supporting Information

A microparticulate based formulation to protect therapeutic enzymes from proteolytic digestion: phenylalanine ammonia lyase as case study

Irene Pereira de Sousa[1], Charlotte Gourmel[1], Olena Berkovska[1], Michael Burger[1], Jean-Christophe Leroux[1]*

[1]ETH Zurich, Department of Chemistry and Applied Biosciences, Institute of Pharmaceutical Sciences, Vladimir-Prelog-Weg 3, 8093 Zurich, Switzerland.

* Prof. Dr. Jean-Christophe Leroux

ETH Zurich, Department of Chemistry and Applied Biosciences

Vladimir-Prelog-Weg 3, 8093 Zurich, Switzerland

E-mail: jleroux@ethz.ch

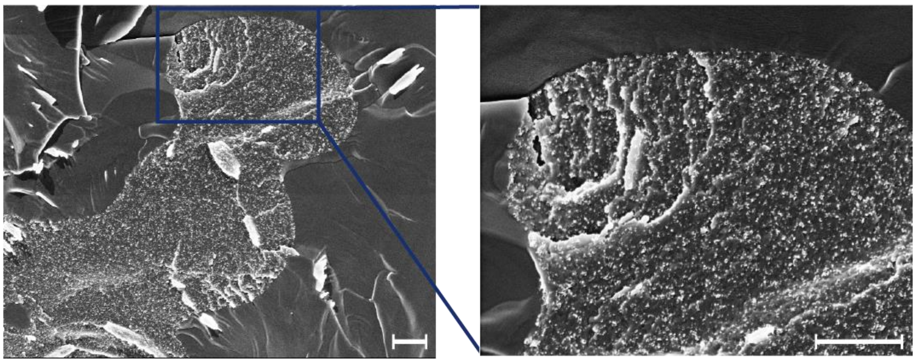

Figure S1. Cryo-SEM images of MSP-l without freeze-drying step. Scale bars represent 1 µm.

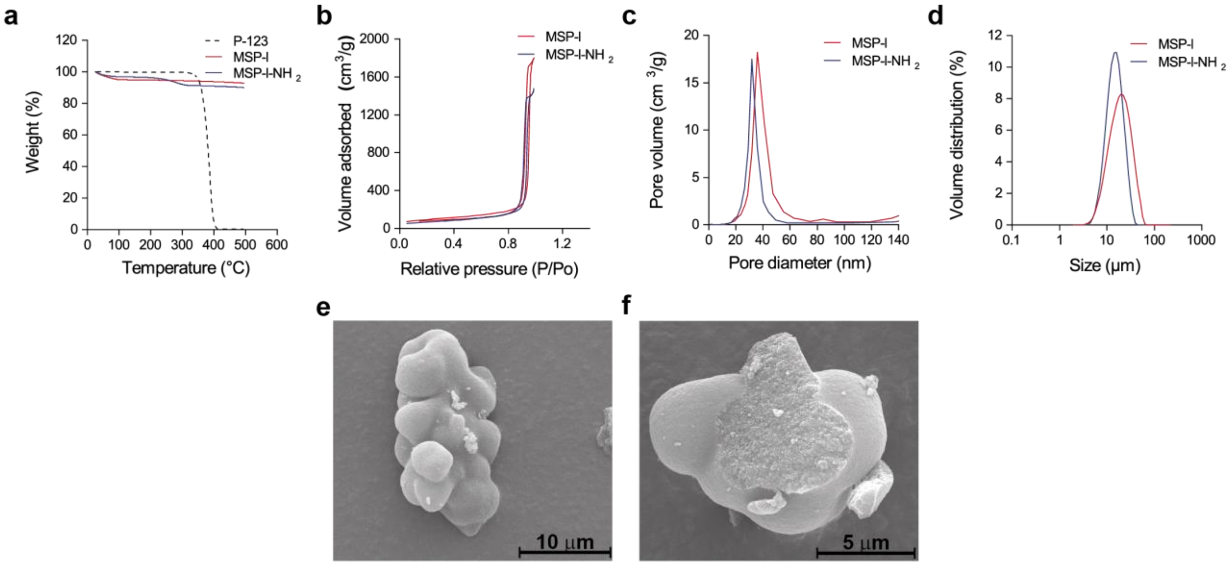

Figure S2. (a) TGA profiles of P-123, MSP-l and MSP-l-NH_2. Nitrogen sorption isotherms (b), pore size distribution (c), and particle size distribution (d) of MSP-l and MSP-l-NH_2. SEM images of an intact MSP-l-NH_2 (e) and a damaged particle exposing the porous inner structure (f).

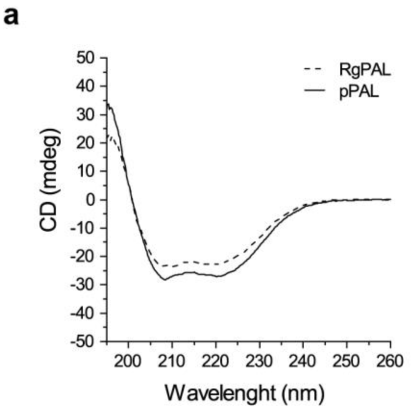

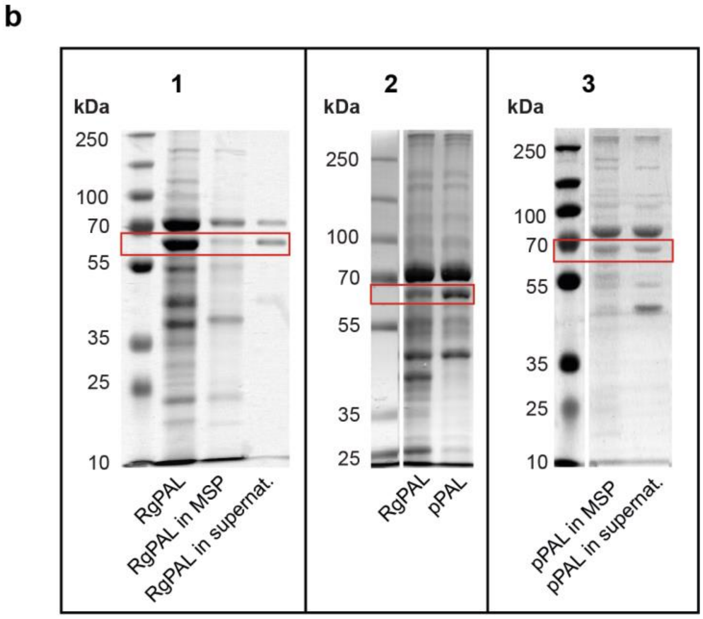

c

	RgPAL	pPAL
K_m (mM)	0.61 ± 0.08	0.64 ± 0.11
V_{max} (mM/min)	0.019 ± 0.001	0.026 ± 0.01
Specific activity (μmol/min/mg)	0.74 ± 0.19	1.15 ± 0.25

Figure S3. (a) Circular dichroism spectra of PAL before (RgPAL) and after (pPAL) purification. (b1) SDS-PAGE of RgPAL, RgPAL loaded in MSP and of the free fraction. (b2) SDS-PAGE of RgPAL and pPAL. (b3) SDS-PAGE of pPAL loaded in MSP and of the free fraction. The area corresponding to the molecular weight of PAL's monomers is framed in red. The reported figures are contrast adjusted cropped images of the original gels that have been combined to make the figure clearer for the reader; full length gels are reported in Figures S12 (for b1), S13 (for b2) and S14 (for b3). (c) Comparison of activity and kinetic constants of RgPAL and pPAL.

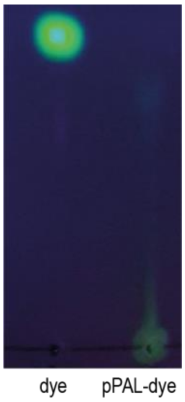

Figure S4. TLC of the dye (BDP FL NHS) and of the pPAL-dye conjugate.

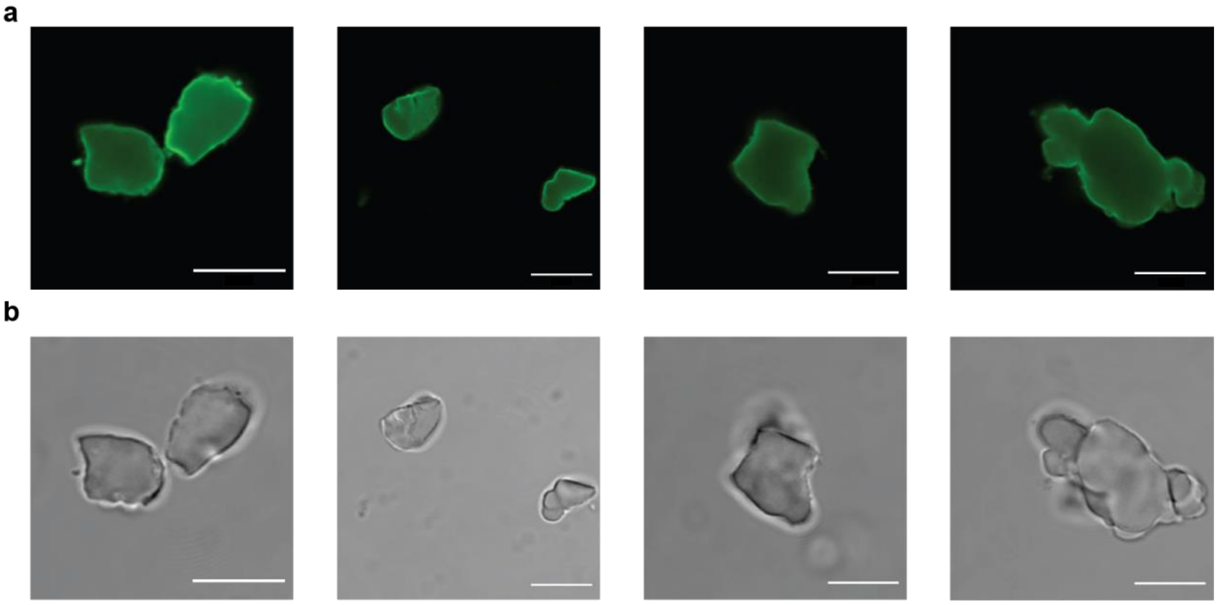

Figure S5. Additional representative confocal microscopy images of several MSP-PAL loaded with fluorescent PAL (fluorescence channel (a) and bright field (b)). Images have been collected by z-stacking and represent the central axial section of the particles. Scale bars represent 10 µm.

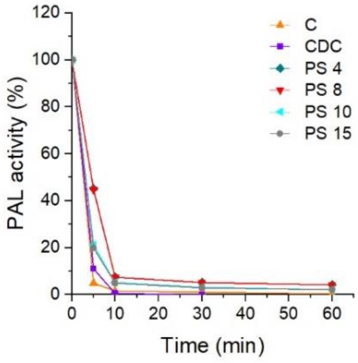

Figure S6. Activity of MSP-PAL coated with 1 layer of chitosan (C), chitosan-dextran sulfate-chitosan (CDC), PAH and polystyrene in succession to form 4 layers (PS 4), 8 layers (PS 8), 10 layers (PS 10) and 15 layers (PS 15) in presence of trypsin 0.4 mg/mL.

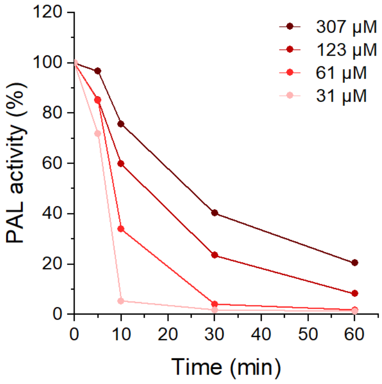

Figure S7. Activity of pPAL in presence of trypsin (0.4 mg/mL) and increasing concentrations of aprotinin (31, 61, 123, or 307 µM).

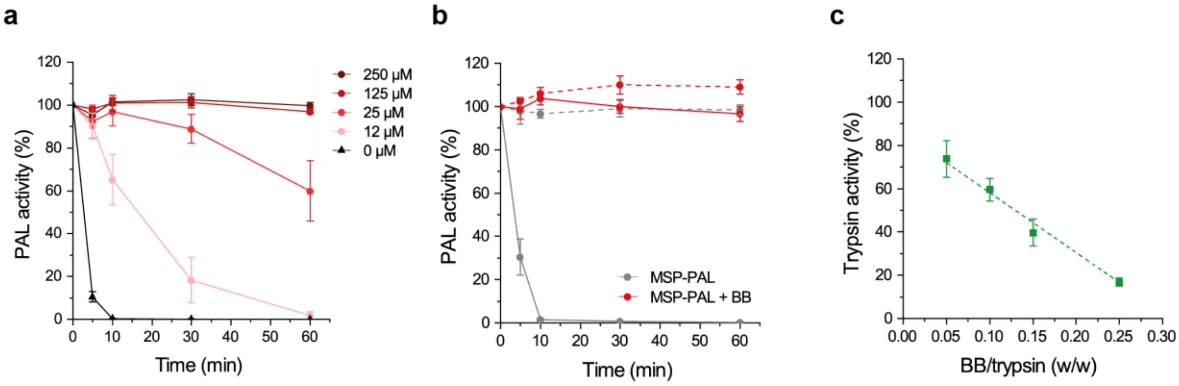

Figure S8. (a) Activity of pPAL in presence of trypsin (0.4 mg/mL) and increasing concentrations of BB (0, 12, 25, 125, 250 µM). (b) Activity of MSP-PAL and MSP-PAL mixed with BB (125 µM) in SIF (dotted lines) and in the presence of trypsin 0.4 mg/mL (solid lines). (c) Remaining trypsin activity after 5 min incubation with increasing concentrations of BB. Data represent the mean ± SD (n = 3).

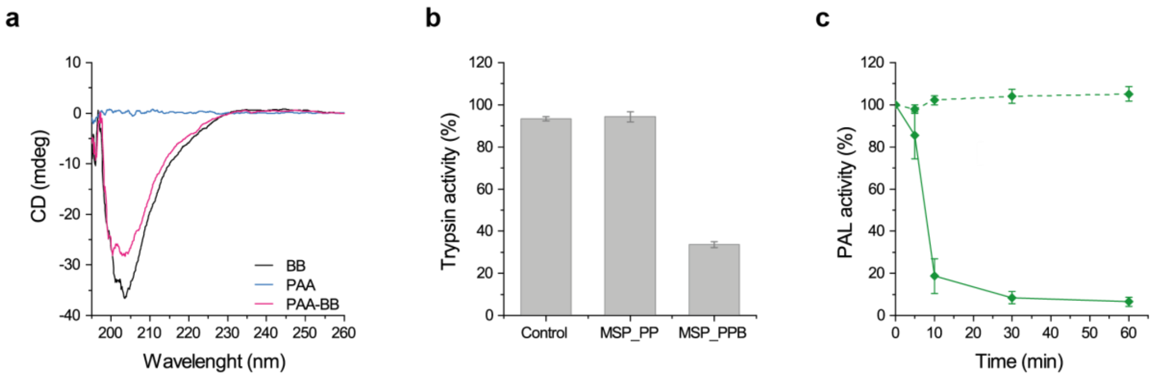

Figure S9. (a) Circular dichroism spectra of BB, PAA, and PAA-BB. (b) Trypsin activity after 5 min incubation with buffer (control), MSP coated with PAH and PAA (MSP_PP) and MSP coated with PAH and PAA-BB (MSP_PPB). (c) Activity of MSP-PAL coated with PAH and PAA/BB mixture in SIF (dotted lines) and in the presence of trypsin 0.4 mg/mL (solid lines). Data represent the mean ± SD (n = 3).

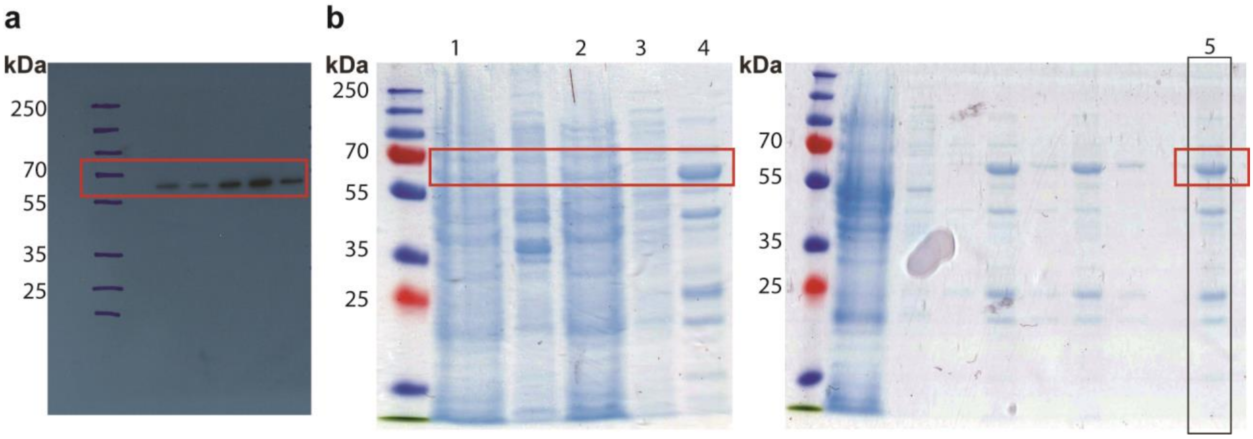

Figure S10. (a) Western Blot stained for the hexahistidine tag of pET His6 TEV LIC colony expressing AvPAL. (b) SDS-PAGE of the AvPAL expression steps: cell lysate supernatant (1), Ni-NTA flow-through (2), Ni-NTA wash with imidazole 10 mM (3), elution with imidazole 250 mM (4), AvPAL after dialysis blotted in a separate gel the consecutive day of the expression (5). The area corresponding to the molecular weight of PAL's monomers is framed in red. The parts of the SDS-PAGE gels that are not labeled by a number represent samples that are not relevant for the outlined work.

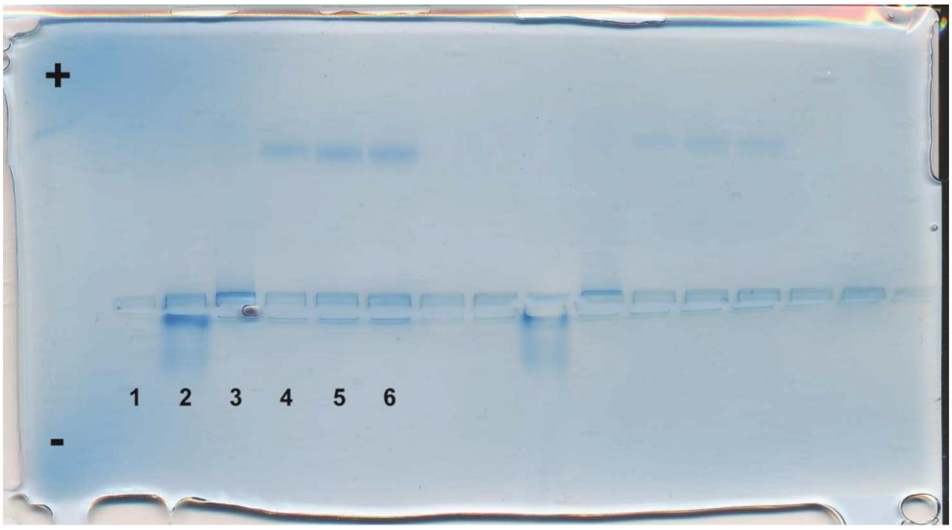

Figure S11. Full length agarose gel electrophoresis image of PAA (1), BB (2), PAA/BB physical mixture (3), and PAA-BB from 3 independent syntheses (4, 5, 6) at pH 4.1 in acetate buffer. The parts of the gel that are not labeled by a number represent samples that are not relevant for the outlined work.

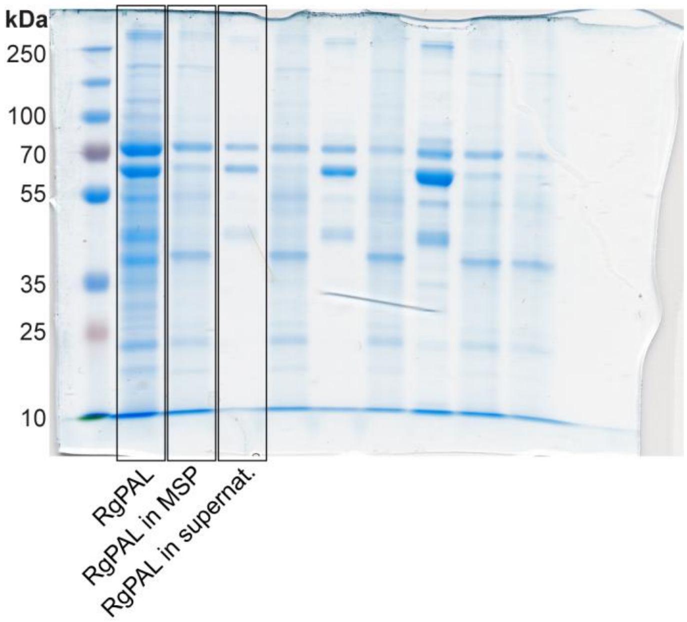

Figure S12. Full length SDS-PAGE gel of RgPAL, RgPAL loaded in MSP and of the free fraction. The parts of the gel that are not framed in black represent samples that are not relevant for the outlined work.

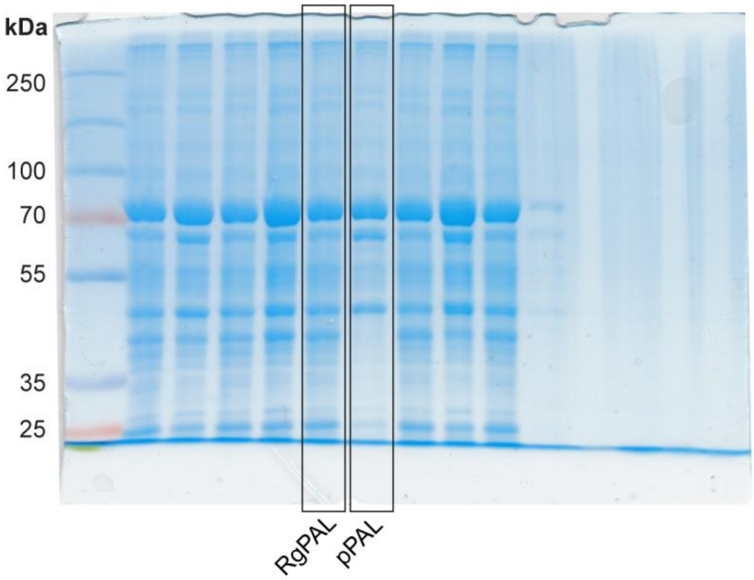

Figure S13. Full length SDS-PAGE gel of of RgPAL and pPAL. The parts of the gel that are not framed in black represent samples that are not relevant for the outlined work.

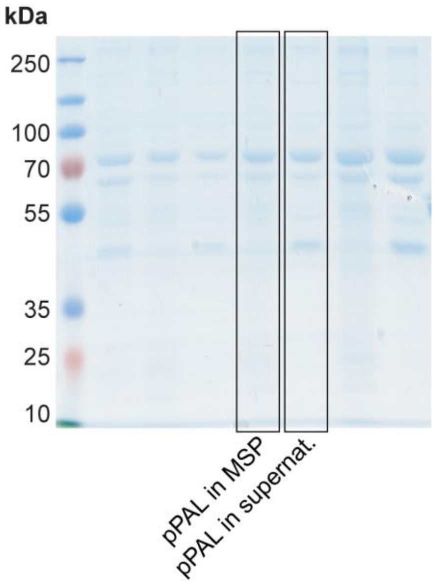

Figure S14. Full length SDS-PAGE gel of pPAL loaded in MSP and of the free fraction. The parts of the gel that are not framed in black represent samples that are not relevant for the outlined work.

Table S1. Characterization of MSP-l-NH_2 in terms of specific surface area, pore volume, pore size, particle size, span and amount of conjugated primary amino groups.

Characteristic	MSP-l-NH_2
**Specific surface area [m^2/g]**	284
**Pore volume, adsorption [cm^3/g]**	2.3
**Pore size, adsorption [nm]**	32.0
**Particle size [µm]**	13.8
**Span**	1.1
**Amount of primary amino groups [µmol/g]**	198 ± 13

Table S2. Encapsulation efficacy (ee) and drug loading (dl) of RgPAL or AvPAL in MSP-s, MSP-l, and MSP-l-NH_2 performed at a PAL/MSP mass ratio of 1:10. Data are expressed as mean ± SD (n = 3).

Sample	ee [%]	dl [%]
**AvPAL/MSP-s**	24.6 ± 12.6	2.9 ± 1.8
**AvPAL/MSP-l**	81.4 ± 3.5	8.5 ± 1.6
**AvPAL/MSP-l-NH_2**	86.9 ± 3.7	9.1 ± 1.8
**RgPAL/MSP-s**	18.6 ± 2.3	2.3 ± 0.3
**RgPAL/MSP-l**	54.7 ± 6.1	5.9 ± 0.5

Table S3. Encapsulation efficacy (ee), drug loading (dl), and activity per mg RgPAL of RgPAL in MSP-l at different PAL/MSP mass ratios. Data represent the mean ± SD (n = 3).

PAL/MSP [w/w]	ee [%]	dl [%]	Activity [IU/mg_{PAL}]
**1:10**	58.4 ± 3.4	5.9 ± 0.5	0.69 ± 0.11
**2:10**	63.4 ± 1.4	12.9 ± 1.5	0.44 ± 0.06
**4:10**	64.9 ± 5.1	26.3 ± 1.8	0.22 ± 0.04

Table S4. Freeze-drying steps for cryo-SEM sample preparation. The process was carried out in a BAF060 (Leica, Vienna) after freeze fracture. At the end of the process the samples were coated with tungsten *via* electron evaporation (5 nm, 45 ° angle with sample rotating at 40 rpm).

Steps	T_{start} [°C]	T_{end} [°C]	Ramp [°C/h]	Hold at T_{end} [h]
1	-120	-115	30	1
2	-115	-110	30	1
3	-110	-100	30	1
4	-100	-90	30	1
5	-90	-80	30	1
6	-80	20	30	-

Table S5. DNA sequence of the plasmid pET His6 TEV LIC (1B) containing the DNA fragment encoding for AvPAL. In the sequence are highlighted: T7 promoter (blue), starting methionine (red) hexahistidine tag (light blue) HindIII restriction site (green), TEV cleavage site (orange), AvPAL sequence (italic, double mutation in bold), stop codon (pink) and XhoI restriction site (violet).

**DNA sequence**
TGGCGAATGGGACGCGCCCTGTAGCGGCGCATTAAGCGCGGCGGGTGTGGTGGTTACGCGCAGCGTGACC
GCTACACTTGCCAGCGCCCTAGCGCCCGCTCCTTTCGCTTTCTTCCCTTCCTTTCTCGCCACGTTCGCCGGC
TTTCCCCGTCAAGCTCTAAATCGGGGGCTCCCTTTAGGGTTCCGATTTAGTGCTTTACGGCACCTCGACCCC
AAAAAACTTGATTAGGGTGATGGTTCACGTAGTGGGCCATCGCCCTGATAGACGGTTTTTCGCCCTTTGACG
TTGGAGTCCACGTTCTTTAATAGTGGACTCTTGTTCCAAACTGGAACAACACTCAACCCTATCTCGGTCTATT
CTTTTGATTTATAAGGGATTTTGCCGATTTCGGCCTATTGGTTAAAAAATGAGCTGATTTAACAAAAATTTAAC
GCGAATTTTAACAAACTAGTAACGTTTACAATTTCAGGTGGCACTTTTCGGGGAAATGTGCGCGGAACCCCTA
TTTGTTTATTTTTCTAAATACATTCAAATATGTATCCGCTCATGAATTAATTCTTAGAAAAACTCATCGAGCATC
AAATGAAACTGCAATTTATTCATATCAGGATTATCAATACCATATTTTTGAAAAAGCCGTTTCTGTAATGAAGGA
GAAAACTCACCGAGGCAGTTCCATAGGATGGCAAGATCCTGGTATCGGTCTGCGATTCCGACTCGTCCAACA
TCAATACAACCTATTAATTTCCCCTCGTCAAAAATAAGGTTATCAAGTGAGAAATCACCATGAGTGACGACTGA
ATCCGGTGAGAATGGCAAAAGTTTATGCATTTCTTTCCAGACTTGTTCAACAGGCCAGCCATTACGCTCGTCA
TCAAAATCACTCGCATCAACCAAACCGTTATTCATTCGTGATTGCGCCTGAGCGAGACGAAATACGCGATCG
CTGTTAAAAGGACAATTACAAACAGGAATCGAATGCAACCGGCGCAGGAACACTGCCAGCGCATCAACAATG
TTTTCACCTGAATCAGGATATTCTTCTAATACCTGGAATGCTGTTTTCCCGGGGATCGCAGTGGTGAGTAACC
ATGCATCATCAGGAGTACGGATAAAATGCTTGATGGTCGGAAGAGGCATAAATTCCGTCAGCCAGTTTAGTC
TGACCATCTCATCTGTAACATCATTGGCAACGCTACCTTTGCCATGTTTCAGAAACAACTCTGGCGCATCGGG
CTTCCCATACAATCGATAGATTGTCGCACCTGATTGCCCGACATTATCGCGAGCCCATTTATACCCATATAAA
TCAGCATCCATGTTGGAATTTAATCGCGGCCTAGAGCAAGACGTTTCCCGTTGAATATGGCTCATAACACCCC
TTGTATTACTGTTTATGTAAGCAGACAGTTTTATTGTTCATGACCAAAATCCCTTAACGTGAGTTTTCGTTCCA
CTGAGCGTCAGACCCCGTAGAAAAGATCAAAGGATCTTCTTGAGATCCTTTTTTTCTGCGCGTAATCTGCTGC
TTGCAAACAAAAAAACCACCGCTACCAGCGGTGGTTTGTTTGCCGGATCAAGAGCTACCAACTCTTTTTCCGA
AGGTAACTGGCTTCAGCAGAGCGCAGATACCAAATACTGTCCTTCTAGTGTAGCCGTAGTTAGGCCACCACT
TCAAGAACTCTGTAGCACCGCCTACATACCTCGCTCTGCTAATCCTGTTACCAGTGGCTGCTGCCAGTGGCG
ATAAGTCGTGTCTTACCGGGTTGGACTCAAGACGATAGTTACCGGATAAGGCGCAGCGGTCGGGCTGAACG
GGGGGTTCGTGCACACAGCCCAGCTTGGAGCGAACGACCTACACCGAACTGAGATACCTACAGCGTGAGCT
ATGAGAAAGCGCCACGCTTCCCGAAGGGAGAAAGGCGGACAGGTATCCGGTAAGCGGCAGGGTCGGAACA
GGAGAGCGCACGAGGGAGCTTCCAGGGGGAAACGCCTGGTATCTTTATAGTCCTGTCGGGTTTCGCCACCT
CTGACTTGAGCGTCGATTTTTGTGATGCTCGTCAGGGGGGCGGAGCCTATGGAAAAACGCCAGCAACGCGG
CCTTTTTACGGTTCCTGGCCTTTTGCTGGCCTTTTGCTCACATGTTCTTTCCTGCGTTATCCCCTGATTCTGTG
GATAACCGTATTACCGCCTTTGAGTGAGCTGATACCGCTCGCCGCAGCCGAACGACCGAGCGCAGCGAGTC
AGTGAGCGAGGAAGCGGAAGAGCGCCTGATGCGGTATTTTCTCCTTACGCATCTGTGCGGTATTTCACACCG
CATATATGGTGCACTCTCAGTACAATCTGCTCTGATGCCGCATAGTTAAGCCAGTATACACTCCGCTATCGCT
ACGTGACTGGGTCATGGCTGCGCCCCGACACCCGCCAACACCCGCTGACGCGCCCTGACGGGCTTGTCTG
CTCCCGGCATCCGCTTACAGACAAGCTGTGACCGTCTCCGGGAGCTGCATGTGTCAGAGGTTTTCACCGTC
ATCACCGAAACGCGCGAGGCAGCTGCGGTAAAGCTCATCAGCGTGGTCGTGAAGCGATTCACAGATGTCTG
CCTGTTCATCCGCGTCCAGCTCGTTGAGTTTCTCCAGAAGCGTTAATGTCTGGCTTCTGATAAAGCGGGCCA
TGTTAAGGGCGGTTTTTTCCTGTTTGGTCACTGATGCCTCCGTGTAAGGGGGATTTCTGTTCATGGGGGTAAT
GATACCGATGAAACGAGAGAGGATGCTCACGATACGGGTTACTGATGATGAACATGCCCGGTTACTGGAAC
GTTGTGAGGGTAAACAACTGGCGGTATGGATGCGGCGGGACCAGAGAAAAATCACTCAGGGTCAATGCCAG
CGCTTCGTTAATACAGATGTAGGTGTTCCACAGGGTAGCCAGCAGCATCCTGCGATGCAGATCCGGAACATA
ATGGTGCAGGGCGCTGACTTCCGCGTTTCCAGACTTTACGAAACACGGAAACCGAAGACCATTCATGTTGTT
GCTCAGGTCGCAGACGTTTTGCAGCAGCAGTCGCTTCACGTTCGCTCGCGTATCGGTGATTCATTCTGCTAA
CCAGTAAGGCAACCCCGCCAGCCTAGCCGGGTCCTCAACGACAGGAGCACGATCATGCGCACCCGTGGGG
CCGCCATGCCGGCGATAATGGCCTGCTTCTCGCCGAAACGTTTGGTGGCGGGACCAGTGACGAAGGCTTGA
GCGAGGGCGTGCAAGATTCCGAATACCGCAAGCGACAGGCCGATCATCGTCGCGCTCCAGCGAAAGCGGT
CCTCGCCGAAAATGACCCAGAGCGCTGCCGGCACCTGTCCTACGAGTTGCATGATAAAGAAGACAGTCATAA
GTGCGGCGACGATAGTCATGCCCCGCGCCCACCGGAAGGAGCTGACTGGGTTGAAGGCTCTCAAGGGCAT
CGGTCGAGATCCCGGTGCCTAATGAGTGAGCTAACTTACATTAATTGCGTTGCGCTCACTGCCCGCTTTCCA
GTCGGGAAACCTGTCGTGCCAGCTGCATTAATGAATCGGCCAACGCGCGGGGAGAGGCGGTTTGCGTATTG
GGCGCCAGGGTGGTTTTTCTTTTCACCAGTGAGACGGGCAACAGCTGATTGCCCTTCACCGCCTGGCCCTG
AGAGAGTTGCAGCAAGCGGTCCACGCTGGTTTGCCCCAGCAGGCGAAAATCCTGTTTGATGGTGGTTAACG
GCGGGATATAACATGAGCTGTCTTCGGTATCGTCGTATCCCACTACCGAGATATCCGCACCAACGCGCAGCC
CGGACTCGGTAATGGCGCGCATTGCGCCCAGCGCCATCTGATCGTTGGCAACCAGCATCGCAGTGGGAAC
GATGCCCTCATTCAGCATTTGCATGGTTTGTTGAAAACCGGACATGGCACTCCAGTCGCCTTCCCGTTCCGC
TATCGGCTGAATTTGATTGCGAGTGAGATATTTATGCCAGCCAGCCAGACGCAGACGCGCCGAGACAGAACT
TAATGGGCCCGCTAACAGCGCGATTTGCTGGTGACCCAATGCGACCAGATGCTCCACGCCCAGTCGCGTAC
CGTCTTCATGGGAGAAAATAATACTGTTGATGGGTGTCTGGTCAGAGACATCAAGAAATAACGCCGGAACAT
TAGTGCAGGCAGCTTCCACAGCAATGGCATCCTGGTCATCCAGCGGATAGTTAATGATCAGCCCACTGACGC

GTTGCGCGAGAAGATTGTGCACCGCCGCTTTACAGGCTTCGACGCCGCTTCGTTCTACCATCGACACCACCA
CGCTGGCACCCAGTTGATCGGCGCGAGATTTAATCGCCGCGACAATTTGCGACGGCGCGTGCAGGGCCAG
ACTGGAGGTGGCAACGCCAATCAGCAACGACTGTTTGCCCGCCAGTTGTTGTGCCACGCGGTTGGGAATGT
AATTCAGCTCCGCCATCGCCGCTTCCACTTTTTCCCGCGTTTTCGCAGAAACGTGGCTGGCCTGGTTCACCA
CGCGGGAAACGGTCTGATAAGAGACACCGGCATACTCTGCGACATCGTATAACGTTACTGGTTTCACATTCA
CCACCCTGAATTGACTCTCTTCCGGGCGCTATCATGCCATACCGCGAAAGGTTTTGCGCCATTCGATGGTGT
CCGGGATCTCGACGCTCTCCCTTATGCGACTCCTGCATTAGGAAGCAGCCCAGTAGTAGGTTGAGGCCGTT
GAGCACCGCCGCCGCAAGGAATGGTGCATGCAAGGAGATGGCGCCCAACAGTCCCCCGGCCACGGGGCCT
GCCACCATACCCACGCCGAAACAAGCGCTCATGAGCCCGAAGTGGCGAGCCCGATCTTCCCCATCGGTGAT
GTCGGCGATATAGGCGCCAGCAACCGCACCTGTGGCGCCGGTGATGCCGGCCACGATGCGTCCGGCGTAG
AGGATCGAGATCTCGATCCCGCGAAAT**TAATACGACTCACTATAGGG**GAATTGTGAGCGGATAACAATTCCC
CTCTAGAAATAATTTTGTTTAACTTTAAGAAGGAGATATACC**ATG**GGTTCTTCT**CACCATCACCATCACCAT**AT
TGATCCCTTCACC**AAGCTT**GCGGATCCG**GAAAACCTGTATTTTCAG***ATGAAAACCCTGAGCCAGGCACAGA*
GCAAAACCAGCAGCCAGCAGTTTAGCTTTACCGGCAATAGCAGCGCAAATGTGATTATTGGTAATCAGAAAC
TGACCATCAATGATGTTGCACGTGTTGCCCGTAATGGCACCCTGGTTAGCCTGACCAATAATACCGATATTCT
GCAGGGTATTCAGGCCAGCTGTGATTATATCAATAATGCAGTTGAAAGCGGTGAACCGATTTATGGTGTTACC
AGCGGTTTTGGTGGTATGGCAAATGTTGCAATTAGCCGTGAACAGGCAAGCGAACTGCAGACCAATCTGGTT
TGGTTTCTGAAAACCGGTGCAGGTAATAAACTGCCGCTGGCAGATGTTCGTGCAGCAATGCTGCTGCGTGCA
AATAGCCACATGCGTGGTGCAAGCGGTATTCGTCTGGAACTGATTAAACGCATGGAAATCTTTCTGAATGCC
GGTGTTACCCCGTATGTTTATGAATTTGGTAGCATTGGTGCCAGCGGTGATCTGGTTCCGCTGAGCTATATTA
CCGGTAGCCTGATTGGCCTGGACCCGAGCTTTAAAGTTGATTTTAATGGCAAAGAAATGGACGCACCGACCG
CACTGCGTCAGCTGAATCTGAGTCCGCTGACCCTGCTGCCGAAAGAAGGTCTGGCAATGATGAATGGCACC
AGCGTTATGACCGGTATTGCAGCAAATTGTGTTTATGATACCCAGATTCTGACCGCAATTGCAATGGGTGTTC
ATGCACTGGATATTCAGGCACTGAATGGTACAAATCAGAGCTTTCATCCGTTTATCCATAACAGCAAACCGCA
TCCGGGTCAGCTGTGGGCAGCAGATCAGATGATTAGCCTGCTGGCCAATAGCCAGCTGGTTCGTGATGAAC
TGGATGGTAAACATGATTATCGTGATCATGAACTGATCCAGGATCGTTATAGCCTGCGTTGTCTGCCGCAGTA
TCTGGGTCCGATTGTTGATGGTATTAGCCAGATTGCCAAACAAATCGAAATTGAGATTAACAGCGTTACCGAT
AACCCGCTGATTGATGTTGATAATCAGGCAAGCTATCATGGTGGTAATTTTCTGGGTCAGTATGTTGGTATGG
GTATGGATCATCTGCGCTATTATATCGGTCTGCTGGCAAAACATCTGGATGTTCAGATTGCACTGCTGGCATC
ACCGGAATTTAGCAATGGTCTGCCTCCGAGTCTGCTGGGTAATCGTGAACGTAAAGTTAATATGGGTCTGAA
AGGTCTGCAGATTTGCGGTAATAGCATTATGCCGCTGCTGACCTTTTATGGTAATAGTATTGCAGATCGTTTT
CCGACCCATGCCGAACAGTTTAACCAGAATATTAACAGCCAGGGTTATACCAGCGCAACCCTGGCACGTCGT
AGCGTTGATATTTTTCAGAATTATGTTGCCATTGCCCTGATGTTTGGTGTTCAGGCAGTTGATCTGCGTACCT
*ACAAAAAAACCGGTCATTATGATGCACGTGCC**AGC**CTGTCACCGGCAACCGAACGTCTGTATAGCGCAGTTC*
GTCATGTTGTTGGTCAGAAACCGACCTCAGATCGTCCGTATATTTGGAATGATAATGAACAGGGTCTGGATGA
ACATATTGCACGTATTAGTGCAGATATTGCAGCCGGTGGTGTTATTGTTCAGGCCGTTCAGGACATTCTGCC
*G**AGC**CTGCAT***TAATAA**AA**CTCGAG**CACCACCACCACCACCACTGAGATCCGGCTGCTAACAAAGCCCGAAA
GGAAGCTGAGTTGGCTGCTGCCACCGCTGAGCAATAACTAGCATAACCCCTTGGGGCCTCTAAACGGGTCT
TGAGGGGTTTTTTGCTGAAAGGAGGAACTATATCCGGAT
